# Asking the generalist – evaluation of a General Practice rounding and consult service

**DOI:** 10.1186/s12875-024-02353-0

**Published:** 2024-04-16

**Authors:** Katharina Schmalstieg-Bahr, Sophia MacDonald, Nadine Pohontsch, Sebastian Debus, Martin Scherer

**Affiliations:** 1 Department of General Practice and Primary Care, University Medical Center Hamburg-Eppendorf, Martinistrasse 52, Building W37, 20246 Hamburg, Germany; 2 Department of Vascular Medicine, University Medical Center Hamburg-Eppendorf, Martinistrasse 52, 20246 Hamburg, Germany

**Keywords:** General practice, Multimorbidity, Interdisciplinary rounds, Co-management, Consults, Interdisciplinary communication, Hospital care

## Abstract

**Background:**

Vascular surgery patients admitted to the hospital are often multimorbid. In case of questions regarding chronic medical problems different specialties are consulted, which leads to a high number of treating physicians and possibly contradicting recommendations. The General Practitioner´s (GP) view could minimize this problem. However, it is unknown for which medical problems a GP would be consulted and if regular GP-involvement during rounds would be considered helpful by the specialists. The aim of this study was to establish and describe a General Practice rounding service (GP-RS), to evaluate if the GP-RS is doable in a tertiary care hospital and beneficial to the specialists and to explore GP-consult indications.

**Methods:**

The GP-RS was established as a pilot project. Between June-December 2020, a board-certified GP from the Department of General Practice and Primary Care, University Medical Center Hamburg-Eppendorf (UKE) joined the vascular surgery team (UKE) once-weekly on rounds. The project was evaluated using a multi-methods approach: semi-structured qualitative interviews were conducted with vascular surgery physicians that had either participated in the GP-RS (G1), had not participated (G2), other specialists usually conducting consults on the vascular surgery floor (G3) and with the involved GP (G4). Interviews were analyzed using Kuckartz’ qualitative content analysis. In addition, two sets of quantitative data were descriptively analyzed focusing on the reasons for a GP-consult: one set from the GP-RS and one from an established, conventional “as needed” GP-consult service.

**Results:**

15 interviews were conducted. Physicians perceived the GP-RS as beneficial, especially for surgical patients (G1-3). Optimizing medication, avoiding unnecessary consults and a learning effect for physicians in training (G1-4) were named as other benefits. Critical voices saw an increased workload through the GP-RS (G1, G3) and some consult requests as too specific for a GP (G1-3). Based on data from 367 vascular surgery patients and 80 conventional GP-consults, the most common reasons for a GP-consult were cardiovascular diseases including hypertension and diabetes.

**Conclusions:**

A GP-RS is doable in a tertiary care hospital. Studies of GP co-management model with closer follow ups would be needed to objectively improve patient care and reduce the overall number of consults.

**Trial registration:**

Not applicable.

**Supplementary Information:**

The online version contains supplementary material available at 10.1186/s12875-024-02353-0.

## Background

Multimorbidity is defined as the presence of two or more, generally chronic, illnesses in one person [1, 2]. The prevalence of multimorbidity in General Practice- as well as in hospitalized patients is high and will most likely continue to increase over the next years [3]. In order to archive optimal care for multimorbid patients, an interdisciplinary approach using the expertise of more than one medical specialty can be helpful. In the hospital setting in Germany and many other countries, this is implemented through consults: the team that is primarily responsible for the patient requests the input from another specialty e.g. recommendations regarding diagnostic work up or therapy options. Hence, the rising prevalence of multimorbid patients has led to an increased involvement of more than one medical specialty in the care of a hospital patient. Especially a consult to Internal Medicine in a surgical patient is common [4, 5]. A study found that up to 86% of all consult requests to the Internal Medicine team in a Spanish hospital came from a surgical department [6]. But Internal Medicine sub-specialization in the fields of e.g. cardiology, pulmonology, nephrology and endocrinology can lead to high number of physicians involved. Although each physician treats the patient according to the guidelines of his or her own specialty, it may not always benefit the patients, as these guideline-based recommendations might be contradicting, increase the number of medications and therefore drug interaction and side effects [7]. The view of a General Practitioner (GP) as a generalist might help to minimize this problem, since GPs often treat multimorbid patients. A systematic review on the prevalence of multimorbidity in GP practices found rates of 75% in patients aged 70 and older [8]. More recent studies found prevalence rates of over 80% in primary care populations [9, 10]. Looking at the most common diagnosis, GPs treat a broad spectrum of medical problems, for example lung-, cardiovascular-, endocrine and musculoskeletal diseases [11] and cover the fields of disease prevention, rehabilitation and psychosocial care [12].

But it is unknown for which question a GP would be consulted by the specialists and if regular GP-involvement during rounds would be considered helpful, possibly more helpful than a conventional “as needed” consult. It is also unknown how specialists usually providing consults feel about the GP-involvement in the care of hospitalized multimorbid patients.

The aim of this study was to (A) establish and describe a General Practice rounding service (GP-RS), (B) to evaluate if the GP-RS is doable in a tertiary care hospital and beneficial to the specialists and (C) to answer the question of what would be the indications for a GP-consult.

## Methods

### Study design

We decided to combine qualitative and quantitative methods in a multi-methods-approach [13] to on the one hand side best fulfill the study objective (B): gaining insight ofthe individual perception regarding the feasibility of a GP-RS from the point of view of involved physicians, theGP and those who did not participate in the pilot project, but would be affected if the pilot project became standard practice. On the other hand we chose this approach to identifying and quantifying indications for a GP-consult (C). While both strands of the study do not inform each other with regard to sampling, data acquisition or analysis, they have equal status in answering the respective research questions and therefore add to the whole picture aimed for by describing the GP-RS (A).

**(A) The General Practice rounding service**

The GP-RS was established in form of a pilot project. Between June and December 2020, a board-certified general practitioner (one person throughout the project) from the Department of General Practice and Primary Care (UKE) joined the vascular surgery team (UKE) once-weekly during rounds. The physician of the vascular surgery team was either board-certified or still in training. Compared to other surgical specialties, vascular surgeons often care for multimorbid patients. This is due to the etiology of peripheral artery disease, making diabetes, hypertension, coronary artery diseases and COPD common comorbidities.

**(B)** Semi-structured interviews were conducted with physicians of the vascular surgery team and specialists that usually provide consults on the floor to evaluate how a once-weekly involvement of a GP during rounds would be perceived. The interviews were qualitatively analyzed.

**(C)** To answer the question regarding GP-consult indications, quantitative patient data was collected during the GP-RS, descriptively analyzed and compared to a dataset from an already established conventional “as needed” consult service of the Department of General Practice and Primary Care (UKE).

### Qualitative data collection and analysis

#### Guideline development, recruiting and interview conduction

The guideline for the semi-structured interviews was developed by SM supported by KSB focusing on three thematic fields: (1) conventional “as needed” consults, (2) the GP-RS, and (3) the comparison of the two and can be found in the appendix. Minimal changes to the guideline were made after two preliminary test interviews regarding the framing of the opening questions and the interviewee’s experience with consults in general. Physicians of four different groups were invited to participate: vascular surgery physicians that either rounded with the GP during the GP-RS (G1) or did not, e.g. had been in the operating room (G2), other specialists that usually answer consult requests from the vascular surgery team (G3) and the GP that participated in the GP-RS (G4). The preliminary interviews were conducted with one G1- and one G2-physician, and the results were included in the analysis.

Potential interviewees were contacted by mail or telephone. Participant selection followed a purposive sampling approach [14]: The aim was to interview all vascular surgery physicians that participated in the interdisciplinary rounds for G1 and as many vascular surgery physicians as needed (to gain data saturation) that did not participate in the interdisciplinary rounds for G2. G3-physicians were recruited among the very large group of usually consulted physicians inviting physicians from various specialties, both sexes and different age groups [14]. The guideline was modified for each group (G1-G4) to consider the knowledge and /or participation in the project per group. The two preliminary test interviews were conducted in March 2022. The remaining 13 interviews were conducted between April and August 2022. Participation was voluntary. Interviewees determined time and place of the interview. Prior to the interview, they were informed of the study goals, gave written informed consent and answered a short demographic questionnaire. No repeat interviews were done. All interviews were conducted by the same interviewer (SM) who took field notes. No other person besides SM and the participant was present during the interview. Interviews were audio-recorded and transcribed verbatim utilizing transcription rules by Dresing and Pehl [15]. Interviews were then pseudonymized for analysis. Transcripts were not handed out except upon request. Interviews were conducted until data saturation as well as an inductive thematic saturation was reached indicating that further data collection and analysis would most likely not result in additional insights into the field of interest [16].

#### Qualitative content analysis

Interviews were analyzed using Kuckartz’ structuring Qualitative Content Analysis [17] to identify underlying themes. This was done by generating codes that were assigned to interview sections. Deductive codes were generated from the interview guideline and inductive codes were generated from the interview material. MAXQDA Analytics Pro 2022 [18] was used to support the analysis. This report was written following the COREQ-Checklist [19], which can found in the appendix.

### Quantitative data collection and analysis

#### Data from the weekly rounding service (pilot project)

The quantitative data from the GP-RS was collected by the GP during rounds (June-December 2020) and transferred to an Excel spreadsheet. Data included the overall number of patients on the floor during rounds and number of patients that were interdisciplinary discussed (either initiated by the vascular surgery physician or GP). For these patients with an indication for a GP-consult the following information was collected: age, sex, main vascular (surgery) diagnosis, reason(s) for interdisciplinary discussion and GP recommendation. The latter was also documented by the GP in the patient´s electronic medical record (EMR), and was automatically included in the discharge letter. In the interest of time, no EMR documentation was done if the GP´s input was minimal, e.g. check lab value. Additional patient information was gathered from the EMR after the patient was discharged and included comorbidities, surgical procedures, length of stay and discharge medication.

#### Data from the established “as needed” consult service

Since January 2015 the Department of General Practice and Primary Care (UKE) has an established cooperation with the adjacent hospital called Facharztklinik Hamburg and offers “as needed” consults to surgical, most often orthopedic patients. The Facharztklinik does not have an Internal Medicine department and is completely independent from the UKE. Consult requests are sent by fax and answered by a board-certified GP (multiple persons over the years) in a timely manner, either by telephone and / or by personal visit. Documentation is done by handwriting on the consult request sheet. After the consult, a copy of the sheet is securely kept in the Department of General Practice and Primary Care (UKE). The original remains in the paper chart of the patient.

The second quantitative dataset was obtained from these consult request sheets and included patients´ age, sex, reason(s) for the consult request, GP´s diagnosis regarding the consult reason and GP´s (treatment) recommendation. No additional information from the patient´s EMR could be obtained, since UKE GPs are only granted access the patient´s paper chart during the consult, but do not have permanent access to the Facharztklinik computer system. The data set included consults done between January 2015 and February 2021.

## Results

### Qualitative results

#### Characteristics of interviewed physicians

All interviewees were UKE physicians. 15 interviews were conducted (G1: *N* = 5; G2: *N* = 4; G3: *N* = 5; G4: *N* = 1) and lasted from 19 to 49 min (mean length: 24.6 min). One interview took place in a private apartment, two in public spaces and 12 on UKE grounds. None of four vascular surgery physicians responsible for the floor at the time of the pilot project refused to participate, namely to round with the GP, and all 4 G1-physicians agreed to an interview (participation rate 100%). One interviewee took part in the GP-RS as a final year medical student and later started to work in the department when being recruited for an interview – initially as a G2-physcian. Although responsibilities differed during the GP-RS and at the time of the interview, a person cannot be isolated from his / her experience. Therefore, the interview was counted as a G1-physican as well. Each G1-physician rounded with the GP one to ten times per person (G1.1: 5x, G1.2: 5x, G1.3: 8x, G1.4: 2x, G1.5:1x). Some physicians who met the criteria for G2 and G3 did not answer e-mails or telephone calls, or stated they were not interested or had no time for an interview, which led to a participation rate of 35% (of contacted physicians) in G2 and 56% in G3. No further information about non-participants was kept. Specialties represented in group 3 were nephrology, cardiology, neurology, rheumatology and pulmonology. Since only on GP was involved in the pilot project, the G4 participation rate was also 100%. Table 1 depicts the interviewees´ demographic characteristics.

**Table 1:** Demographic characteristics interviewed physicians

	Average Age in years (range)	Sex	Average work experience in years (range)	Board certification
G1 (*N* = 5)	30 (28–33)	3 F /2 M	0.5 (0.5-4)	*N* = 0
G2 (*N* = 4)	37.3 (29–46)	1 F /3 M	8 (3–18)	*N* = 1
G3 (*N* = 5)	34.8 (26–38)	1 F /4 M	8.2 (4–11)	*N* = 2
G4 (*N* = 1)	38	1 F	12	*N* = 1

### Impact and challenges of conventional “as needed” consults

Three main themes emerged from the interviews regarding the impact of conventional consults on daily routines: (1) workflow delay on the hospital floor, (2) waiting time until a consult is answered and (3) an increased workload due to consults.

*“That is then already / Yes, when consults get put off, or not done, an extension of the hospital stay becomes possible. Or of any further therapy steps, because you are still waiting for results.”*(G1.3, Pos.28).

“*And with some departments you know that if you request a consult that means you need to order lots and lots of diagnostics before, so to speak, it is satisfactory for the consulted physician*.” (G3.1, Pos.22).

The most frequently mentioned theme by G1-G3 physicians was number (2) and the consequences arising thereof. A connection was drawn between waiting time and the delay of diagnostic tests, procedures, other therapies and patient discharge, which subsequently imposes a burden in form of organizational difficulties on the physician who requested the consult. On the other side, the benefit of a consult were also appreciated in order to optimize patient care.

*“And this is why those [consults] could be more time-consuming, because the appropriate number of tests need to be done. Yes, that is, that is for sure, that can occasionally mean that you got more work, essentially. But what is not bad. I mean, this is why you request a consult, to optimize the therapy or to even start therapy.* (G2.3, Pos.24)

According to physicians in all groups, a problem of conventional consults was that the reason for the consult was often not clearly stated in the consult request. This resulted in difficulties for the consulted physician identifying the underlying task.

*“Because consults are often requested very broadly, especially on non-Internal Medicine floors the consult is not a concrete question. But the consult is then: co-assessment of known disease.”*(G3.1, Pos.69).

Others problem were interdisciplinary communication, for example that no one was available for follow up questions and the sheer number of consults that are constantly requested (G1 and G2). In some cases, consults were only requested for legal purposes or for unspecific symptoms of the patient, thus making it difficult for the requesting physician to contact the correct department for the consult.

*“And then you request a nephro[logy] consult due to legal reasons, because the attendings want that. Well, that has always slightly pissed me off.”* (G1.4, Pos.58).

A complete sub-category overview of challenges of conventional consults is depicted in Figure S1 in the appendix.

### Benefits of the general practice-rounding service

The perspective of the GP as generalist was seen as an advantage (G1-G3), but only for multimorbid surgical patients.

*[…]“ I can imagine that thereby patients are treated a bit more comprehensively. In general, our patients are very […] also most often have internal [medicine] diagnoses That is, I think, it is surely a difference if you asked us or the trauma surgeons that have significantly more younger patients on the floor.”* (G2.2, Pos. 66).

Patients that were already treated on internal medicine floors were thought to benefit less from the GP-RS. Compared to conventional “as needed” consults, the GP-RS was seen as especially helpful due to the fact that some patients’ issues where only identified by the GP during rounds and might have been otherwise overlooked / not lead to a conventional consult request (G1.2, G4), e.g. to offer patients help with smoking cessation, to (re-)check labs or to order a laxative for a patient on opiates. Optimizing (long-term) medications (i.e. diabetes or hypertension medication) was seen as beneficial as GPs might have a broader understanding of these diseases.

*[…]“ therapy optimized, […] in one or another aspect. [An aspect] that maybe one as a vascular surgeon doesn´t always pay attention to during rounds. But maybe also more intentionally also go over, just internal [medicine]-like. The rounds versed, and then also focus on blood pressure, blood sugar etc. Maybe also adjust congestive heart failure medication. Yes, therefore my general feeling is positive in regards to that.”* (G2.3, Pos. 48).

**Fig. 1:**
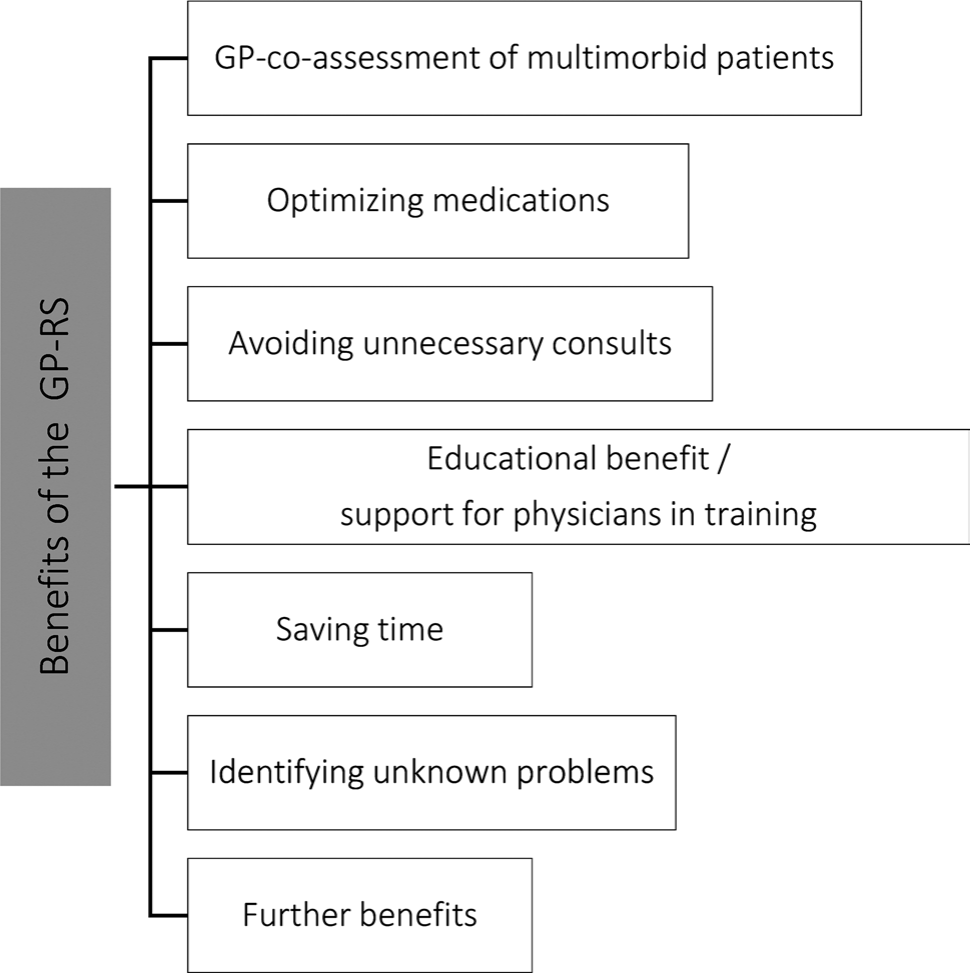
Sub-categories of benefits of the GP-RS

The GP-RS was seen as especially helpful for physicians with less work experience compared to physicians with more work experience (G1-4). An overview of all sub-categories of benefits is depicted in Fig. 1.

### Disadvantages of the general practice-rounding service

**Fig. 2:**
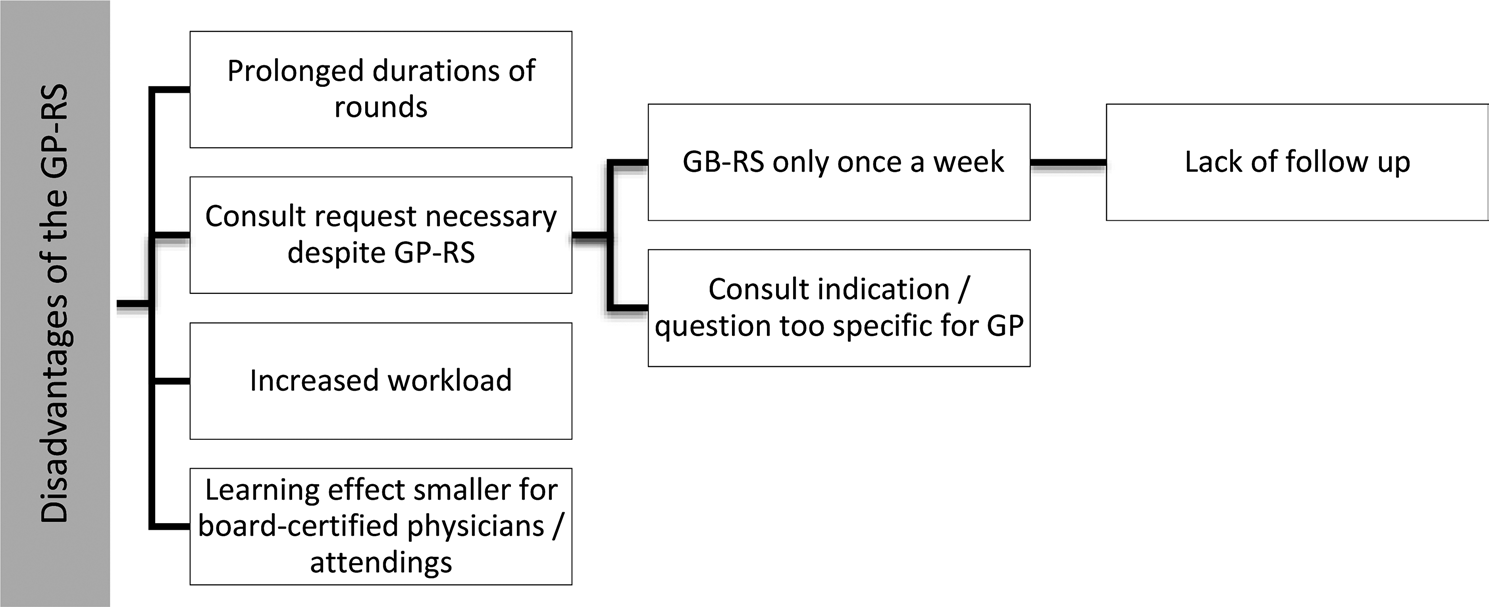
Sub-categories of disadvantages of the GP-RS.

Interviewees identified four main disadvantages of the GP-RS (Fig. 2) which seem partially contradictory to each other, but also contradictory to the identified benefits. On the one hand, it was seen as a disadvantage that a conventional consult needed to be requested anyway because the GP-RS only took place once a week (G1-2, G4). On the other hand, physicians reported that the GP-RS prolonged the rounds (G1) and led to an increased workload (G3).

*“I mean we don’t request a consult for every patient and this, the rounding service, it has the effect, that we have a consultation for every patient. And not just individual cases. And I do think you have to … The advantage of a traditional consult is of course, that you do it specifically [for a specific patient].”* (G3.1, Pos.31).

Two reasons were named for the increased workload: Instead of seeing a single patient due to a conventional consult request, all patients on the floor needed to be seen. And the GP-RS identified problems that would have usually not led to a consult.

*“Well, maybe a small disadvantage would be that usually we are doing surgical rounds, that we look in, look at and that`s it. There with the colleagues we need to look at this, or the labs or this and that. That took a little longer.”* (G1.2, Pos.40).

Another reason for the conventional consult despite the GP-RS was that physicians deemed the question of the consult as too specific for the GP (G1-3).

*“But most consults that are requested are then rather very, very specialty-specific. And I think, without wanting to criticize General Practice in any way, I think that would sometimes just exceed the competency of General Practice.”* (G3.4, Pos.82).

### General practice-rounding service´s influence on conventional consults

There were conflicting statements whether the GP-RS could reduce the number of conventional consults requests from the vascular surgery floor. Physicians in groups G1-G3 thought it was easier and quicker to ask for the GP´s opinion during the GP-RS than to request separate consults for each single patient or each question arising.

*“Yes, positive was, as I said, that that we had were able to directly save a few consults. Directly discuss that with the GP. And then literally had a direct solution during rounds.”* (G1.2, Pos.38).

In contrast, it was stated that the GP-RS took place too seldom to reduce the overall number of consults (G1, G2, G4).

*“Yes, when you do such rounds once a week, you still have on all other days, yes, you must request a consults anyhow.”*(G1.4, Pos.98).

As mentioned before, interviewees from groups G1-G3 thought that some questions might be too specific for a GP and consequently a convention consult request to another specialty was still necessary.

## Quantitative results

### Indications for a GP-consult

#### GP-consult “as needed”

Eighty consult requests from the Facharztklinik were processed during the study period for 75 individual patients (52 female, 28 male). In five cases a re-consults was necessary and patients were seen twice; three of these five patients were female. The mean patient age was 65.5 years (female: 68, male: 61) with a wide range from 26 to 88. These demographic results were different from the results of the 2nd quantitative dataset generated during the GP-RS, since the majority of patients on the vascular surgery floor was male and the average age of female patients differed by 10 years (see below). 16 different GPs conducted the “as needed” consults.

A consult was requested for post-operative patients with newly developed or pre-existing but now exacerbated illnesses. The most common consults reason on the request form was “Co-assessment, please” (*N* = 39) followed by a brief description of the case without any specific request (*N* = 19). Most likely, the intention was to ask for co-assessment. Consult diagnoses documented by the GPs after the consult were widespread and included over 60 different entities. It was possible for one patient to carry more than one diagnosis. The most frequent diagnoses were related to the cardiovascular system (*N* = 55) including cardiac arrhythmia (*N* = 18) and hypertension (*N* = 18). This was followed by diagnosis in the field of pulmonology (*N* = 12), especially COPD (*N* = 3) and pneumonia (*N* = 3). Other fields were less common, such infectious diseases other than pneumonia (*N* = 7) or endocrinology (*N* = 6) with a focus on diabetes (*N* = 4).

#### GP-consult during weekly rounds

On 20 rounding days, the total number of listed vascular surgery patients was 367, including patients that were off the floor for testing or in the operating room, as these were briefly mentioned during rounds. Patients that stayed longer than seven days were counted per rounding day. Forty-four individuals (11 female, 33 male) had an indication for a GP-consult, meaning that these cases were interdisciplinary discussed. Three patients were interdisciplinary discussed more than once (1 patients 4x, 2 patients 2x), which led to 49 patient contacts in relation to 367 patients listed during rounding days (13.4%). In 18 patient contacts (4.9%) the GP´s involvement was significant and her recommendations were documented in the patient´s EMR. The mean age of patients with GP-consult indication was 69.6 years (female: 79, male: 66.9). Usually, consult reasons were pre-existing diseases that exacerbated during the hospital stay, so that further evaluation / diagnostic testing and /or medication adjustment became necessary. An overview is depicted in Table 2.

**Table 2:** Frequency and reasons for GP-consult indication during GP-RS

Diagnosis	Total Number
Blood pressure associates problems (Hypo-/ Hypertension)	11
Diabetes mellitus (NIDDM / IDDM)*	6
Renal insufficiency (with / without dialysis)	6
Nicotine abuse	5
Edema	3
Heparin syringe pump therapy	3
Electrolyte imbalances (hypocalcaemia, hyponatremia)	2
Asthma / COPD	2
Optical hallucinations of unknown origin	1
Hypalbuminemia	1
Dementia	1
Aortic valve stenosis	1
Myelodysplastic syndrome	1
Atrial fibrillation	1
Long-term-I.v.-antibiosis (Myelodysplastic syndrome)	1

* NIDDM / IDDM: (non-) insulin-dependent diabetes mellitus

## Discussion

### Principal results

We established a General Practice rounding service at a tertiary care hospital. Specialists perceived the involvement of a generalist as helpful, but only of multimorbid surgical patients – especially since optimizing medication regimens was one of the reported benefits. Other benefits of the GP-RS were identification of unknown problems, saving time, avoiding unnecessary consults as well as the support and educational benefit for surgical physician in training. Disadvantages of the GP-RS included the prolonged duration of rounds, an increased workload and fact that a conventional consult was still needed. The latter was either due to fact that the once-weekly GP-RS did not allow a close follow up or that specialist deemed the medical problem outside the scope of a GP. However, the results from the two quantitative datasets (from the GP-RS and Facharztklinik) demonstrate a great variety of consult reasons which were well within the scope of a GP.

### Strengths and limitations

This study addresses a relatively new concept: GP-consults, whether in form of a conventional “as needed” consult or in form of a rounding service are not very common. The qualitative analysis reached data saturation as well as an inductive thematic saturation.

In Germany, it is common that the hospital floor – including the rounds – is managed by physicians in training. Therefore, it is was expectable that all G1-physicians were not board-certified. An attending is assigned to supervise them, but he or she might not always be immediately available, e.g. being in the operating room. Our results show that the GP-RS was seen as a way to support physicians in training during rounds and was considered to be less helpful for board-certified physicians. Although this was mentioned in all groups (G1-G4), it is possible that an interview with a G1-board-certified physician would have brought up a different view.

A major limitation is the fact that only one GP and only one vascular surgery team from one hospital were involved. The pilot project was planned with only one GP in order to provide a relatively equal experience to all vascular surgery physicians. It is possible that the involvement of multiple GPs or another team e.g. from a non-tertiary care hospital would have yielded other results. Therefore, the results may not be generally applicable. The GP who took part in the interdisciplinary rounds helped to analyze the data. However, she only had access to pseudonymized data sets and her involvement was minimal (see authors´ contributions). In addition, the time between the last interdisciplinary rounds and the interview was relatively long, which may have affected the interviewees´ recall.

Both quantitative datasets were relatively small. However, the dataset from the Facharztklinik covers a time period of 6 years, so that both quantitative sets together seem to give an accurate picture of why GPs are consulted. No data was acquired regarding the number of consult requests from the vascular surgery team before, during or after the GP-RS.

### Comparison to other studies

Available literature focuses on co-management models between Internal Medicine (not General Practice) and other medical specialties; literature review lacked studies of co-management explicitly with GPs, especially inside the hospital setting. GPs treat a broad spectrum of diseases [11, 12] and due to the ongoing demographic change [20] they are experienced regarding the care of elderly multimorbid patients. Vascular surgery patients often suffered from a broad spectrum of comorbidities; many of these are regularly treated by GPs, like hypertension, coronary artery disease, COPD and diabetes. Studies focusing on interprofessional / interdisciplinary rounds often involve nurses and physicians, and / or include Internal Medicine [21] or other specialties [22, 23], but not General Practice. Even if the results are only indirectly comparable, Vidán et al. demonstrated that early multidisciplinary daily geriatric care led to a reduction in in-hospital mortality and medical complications in elderly patients with hip fracture [24]. And a Spanish study from 2014 aimed to answer the question whether the co-management with Internal Medicine physicians would improve the care of vascular surgery patients [25]. Compared to controls of the year before, study patients showed a higher frequency of identified thrombosis risk factors, an improved use of the correct heparin dose adapted to weight and kidney function, as well as higher percentage of identified comorbidities. No difference was seen regarding the length of the hospital stay. Based on a survey, physicians as well as nursing staff preferred the co-management model over the standard model [25]. However, direct comparison to this study is difficult as in the Spanish study internists where available on a regular daily basis in the emergency department as well as for daily follow-ups, compared to once-weekly rounds.

The quantitative and qualitative results of this study showed that unspecific consult requests are common and cause difficulties for the consulted physician to identify the underlying task. There are no studies focusing on this problem. Instead, literature research yielded studies addressing inappropriate consults [26, 27].

## Conclusions

A General-Practice rounding service on vascular surgery floor in a tertiary care hospital is doable and perceived as helpful by specialist for multimorbid surgical patients and physicians in training. If a GP´s input was requested – whether in form of a conventional “as needed” consult or during the GR-RS – the reason fell well within the GP field of expertise. Whether a GP-RS is able to reduce the overall number of consult request is unclear. Further studies evaluating models with a closer GP follow up e.g. through case conferences and possibly with a control group are necessary. Since Germany as well as many other countries are facing or will face a lack of GPs, involving GPs in hospital medicine carries the potential risk of taking their work capacity away from the ambulatory field. This also needs to be addressed when evaluating future models of comprehensive care for (multimorbid) hospital patients.

### Electronic supplementary material

Below is the link to the electronic supplementary material.

Supplementary Material 1

Supplementary Material 2

Supplementary Material 3

Supplementary Material 4

Supplementary Material 5

Supplementary Material 6

## Data Availability

The datasets generated and/or analyzed during the current study are not publicly available due privacy protection regulations but are available from the corresponding author on reasonable request.

## Abbreviations

COPD: chronic obstructive pulmonary disease
EMR: electronic medical record
GP: General Practice / General Practice
GP-RS: General Practice-rounding service
IDDM: insulin-dependent diabetes mellitus
NIDDM: non-insulin-dependent diabetes mellitus
UKE: University Medical Center Hamburg-Eppendorf
