# Supplementary material for: Asking the generalist – evaluation of a General Practice rounding and consult service
Source: BMC Prim Care. 2024 Apr 16;25:113. doi: 10.1186/s12875-024-02353-0 (PMC11020190; doi:10.1186/s12875-024-02353-0)
Supplement: Supplementary file 2 — Supplementary Material 2 [file 12875_2024_2353_MOESM2_ESM.docx]

**Interview guideline G1**

Introduction

1. Interviewer introduction: name, profession, current workplace (medical student at the Department of General Practice and Primary Care)

2. Overview of the study:

- Interview to capture the subjective experience of the physicians who participated in the pilot project and compare the pilot project to conventional “as needed” consults
- Recording of the interview
- Voluntary participation/withdrawal anytime possible
- Preparation of a transcript. Analysis of pseudonymized data, so that no conclusions can be drawn about the participant during analysis and upon publication
- Passages can be removed upon request at any time, even afterwards
- Ask participant to sign a written consent form
- Thank for participation

Themes:

1. Consultation requests

2. Interdisciplinary rounds

3. Comparison of interdisciplinary rounds and consultation requests

Opening / career path:

“When I ask you questions, you will have as much time as you need to answer them. I will listen to you first and write down notes with regards to the different aspects, which I might come back to later. The aim is to capture your personal experiences and thoughts, and there is no right or wrong answer. We don't know each other yet, maybe you can tell me a few things about yourself, what has been your career path so far?”

Consultation requests:

“I would like to touch on consultation requests first. Can you think of any personal experiences you have had with consultation requests, regardless of the specialty, and tell me about a typical consultation?”

“When you submit a consultation request, what steps do you need to go through?”

Possible follow-up question:

“Which of these specific steps do you think needs the most improvement, and how? “

“What would you like other specialists to do when it comes to consultation requests?”

“How do consult services impact the workflow on the floor? Can you tell me more about it?”

Follow-up questions (by requesting details or paraphrasing), e.g.:

“I would like to go back to the notes I took.”

“You mentioned that... (X). Could you explain it in more detail?”

“You mentioned that... (X). Could you give some more examples?”

“In the situation you described, you noticed that... (X). Do you have any other examples or experiences in this regard?”

“You mentioned the circumstance X. Could you explain it in more details again?”

Interdisciplinary rounds / pilot project:

“If you think back to the time of the interdisciplinary rounds with the GP what comes to your mind spontaneously? Can you tell me about that?”

If participants don't have many or any memories about the project: “Do you remember Katharina Schmalstieg-Bahr, who was regularly on the floor last year? The younger blonde? How was the collaboration with her?”

“What was your impression about the general flow of the interdisciplinary rounds?”

“Would you recommend interdisciplinary rounds to your colleagues? And if so/not, why (not)?”

“Please tell me why you discussed some patients interdisciplinary and others not.”

“In what way did your attitude towards interdisciplinary rounds change during the course of the project?”

“Have you ever worked interdisciplinary outside of the pilot project and the consultations? Please describe your experiences.”

“How often have you joined the interdisciplinary rounds?

Follow-up questions: like above.

Comparison of interdisciplinary rounds and consultation requests:

“If you compare traditional consults and regular interdisciplinary rounds, what thoughts come to your mind?”

“How do you rate the effectiveness of the pilot project compared to consults in terms of patient care on your floor?”

“How would you change the interdisciplinary collaboration if you could?

“If we're talking about the pilot project here, does that trigger anything else for you? You are welcome to tell me what's on your mind.”

If not mentioned yet:

“Which model would you prefer in the future? Why?”

“Was there something missing for you during the interdisciplinary rounds, or would you do something differently in the future?”

Conclusion:

“Has anything else come to your mind in conjunction with interdisciplinary rounds or consult requests that we haven't discussed yet?”

“I thank you for your time and effort.”
