# Supplementary material for: Asking the generalist – evaluation of a General Practice rounding and consult service
Source: BMC Prim Care. 2024 Apr 16;25:113. doi: 10.1186/s12875-024-02353-0 (PMC11020190; doi:10.1186/s12875-024-02353-0)
Supplement: Supplementary file 3 — Supplementary Material 3 [file 12875_2024_2353_MOESM3_ESM.docx]

**Interview guideline G2**

Introduction

1. Interviewer introduction: name, profession, current workplace (medical student at the Department of General Practice and Primary Care)

2. Overview of the study:

- Outline of the pilot project: Inquire if the participants are familiar with the project in general (have heard of it) or if it needs to be described in more detail.
- Purpose of the interview: to capture the subjective experience of the physicians and compare the pilot project to conventional “as needed” consults
- “Even if you have not participated in the pilot project, I would like to interview you and learn about your experiences with consults and interdisciplinary collaboration.”
- Recording of the interview
- Voluntary participation/withdrawal anytime possible
- Preparation of a transcript. Analysis of pseudonymized data, so that no conclusions can be drawn about the participant during analysis and upon publication
- Passages can be removed upon request at any time, even afterwards
- Ask participant to sign a written consent form
- Thank for participation

Themes:

1. Consultation requests

2. Interdisciplinary rounds

3. Comparison of interdisciplinary rounds and consultation requests

Opening / career path:

“When I ask you questions, you will have as much time as you need to answer them. I will listen to you first and write down notes with regards to the different aspects, which I might come back to later. The aim is to capture your personal experiences and thoughts, and there is no right or wrong answer. We don't know each other yet, maybe you can tell me a few things about yourself, what has been your career path so far?”

Consultation requests:

“I would like to touch on consultation requests first. Can you think of any personal experiences you have had with consultation requests, regardless of the specialty, and tell me about a typical consultation?”

“When you submit a consultation request, what steps do you need to go through?”

Possible follow-up question:

“Which of these specific steps do you think needs the most improvement, and how? “

“What would you like other specialists to do when it comes to consultation requests?”

“You mentioned that (X). Can you imagine that this would have gone differently through interdisciplinary rounds?”

“How do consult services impact the workflow on the floor? Can you tell me more about it?”

“Have you ever worked interdisciplinary outside of consults?”

Follow-up questions (by requesting details or paraphrasing), e.g.:

“I would like to go back to the notes I took.”

“You mentioned that... (X). Could you explain it in more detail?”

“You mentioned that... (X). Could you give some more examples?”

“In the situation you described, you noticed that... (X). Do you have any other examples or experiences in this regard?”

“You mentioned the circumstance X. Could you explain it in more details again?”

Interdisciplinary rounds / pilot project:

“What are your memories of the interdisciplinary pilot project, even if you were not directly involved?

“If nothing comes to mind: Were you aware of the pilot project? Was there any discussion about it within the team? To what extent did you become aware of or learn about the project? (e.g., through colleagues, documentation...?)”

(If not applicable: brief outline the project again by interviewer)

“Maybe there is one aspect, regardless of whether it was positive or negative, that particularly stuck with you. Tell me about it.”

“(Even if you didn't know much about the pilot project) What was your impression of the interdisciplinary rounds during the months they took place?

“How can you imagine integrating this type of collaboration into your daily work routine?”

“Would you recommend interdisciplinary visits to your colleagues? Why (not)?”

“Have you yourself had any experience with interdisciplinary rounds in the past? Tell me about a typical experience.”

Follow-up questions: like above.

Comparison of interdisciplinary rounds and consultation requests:

“If you compare traditional consults and regular interdisciplinary rounds, what thoughts come to your mind?”

“How do you rate the effectiveness of the pilot project compared to consults in terms of patient care on your floor?”

“How would you change the interdisciplinary collaboration if you could?

“If we're talking about the pilot project here, does that trigger anything else for you? You are welcome to tell me what's on your mind.”

If not mentioned yet:

“Which model would you prefer in the future? Why?”

“Was there something missing for you during the interdisciplinary rounds, or would you do something differently in the future?”

Conclusion:

“Has anything else come to your mind in conjunction with interdisciplinary rounds or consult requests that we haven't discussed yet?”

“I thank you for your time and effort.”
