# Supplementary material for: Asking the generalist – evaluation of a General Practice rounding and consult service
Source: BMC Prim Care. 2024 Apr 16;25:113. doi: 10.1186/s12875-024-02353-0 (PMC11020190; doi:10.1186/s12875-024-02353-0)
Supplement: Supplementary file 4 — Supplementary Material 4 [file 12875_2024_2353_MOESM4_ESM.docx]

**Interview guideline G3**

Introduction

1. Interviewer introduction: name, profession, current workplace (medical student at the Department of General Practice and Primary Care)

2. Overview of the study:

- Introduction of the pilot project in broad terms and comparison with consult services
- Goal: Interviewing consulted physicians to inquire about their thoughts about this interdisciplinary model
- “Although you were not involved in the interdisciplinary rounds, I would still like to interview you to hear about your experiences with consultations and your thoughts of this pilot project.”
- Recording of the interview
- Voluntary participation/withdrawal anytime possible
- Preparation of a transcript. Analysis of pseudonymized data, so that no conclusions can be drawn about the participant during analysis and upon publication
- Passages can be removed upon request at any time, even afterwards
- Ask participant to sign a written consent form
- Thank for participation

Themes:

1. Consultation requests

2. Interdisciplinary rounds

3. Comparison of interdisciplinary rounds and consultation requests

Opening / career path:

“When I ask you questions, you will have as much time as you need to answer them. I will listen to you first and write down notes with regards to the different aspects, which I might come back to later. The aim is to capture your personal experiences and thoughts, and there is no right or wrong answer. We don't know each other yet, maybe you can tell me a few things about yourself, what has been your career path so far?”

Consultation requests:

“You surely answer and initiate several consults in your everyday work. Please tell me about a typical consult.”

“When you submit a consultation request, what steps do you need to go through?”

Possible follow-up question:

“Which of these specific steps do you think needs the most improvement, and how? “

“What would you like other specialists to do when it comes to consultation requests?”

“Tell me about a consultation with a multimorbid patient.” (Definition of multimorbidity: more than one chronic illness)

Follow-up questions (by requesting details or paraphrasing), e.g.:

“I would like to go back to the notes I took.”

“You mentioned that... (X). Could you explain it in more detail?”

“You mentioned that... (X). Could you give some more examples?”

“In the situation you described, you noticed that... (X). Do you have any other examples or experiences in this regard?”

“You mentioned the circumstance X. Could you explain it in more details again?”

Interdisciplinary rounds / interdisciplinary collaboration

“Can you provide an example of interdisciplinary collaboration aside from consults?”

“Please tell me about your experiences with interdisciplinary exchange.”

“What comes to mind when you think of interdisciplinary rounds?”

“If you could permanently change something about interdisciplinary collaboration, what would it be?”

Follow-up questions: like above.

Comparison of interdisciplinary rounds and consultation requests:

“When comparing consult requests and interdisciplinary rounds, is there anything else you can think of that we haven't mentioned yet?

“You mentioned that (X). Can you imagine that this would have been different with interdisciplinary rounds?”

There are illnesses that are treated by multiple specialties. What would you think if a patient with disease X is discussed during rounds with a general practitioner instead of primarily submitting a consult request to you?”

(Note: Interview with a pulmonologist: use a patient with COPD as example, endocrinologist: diabetic patient, etc.)

Conclusion:

“Has anything else come to your mind in conjunction with interdisciplinary rounds or consult requests that we haven't discussed yet?”

“I thank you for your time and effort.”
