# Supplementary material for: Asking the generalist – evaluation of a General Practice rounding and consult service
Source: BMC Prim Care. 2024 Apr 16;25:113. doi: 10.1186/s12875-024-02353-0 (PMC11020190; doi:10.1186/s12875-024-02353-0)
Supplement: Supplementary file 5 — Supplementary Material 5 [file 12875_2024_2353_MOESM5_ESM.docx]

**Interview guideline G4**

(Brief) introduction >> Participant knows interviewer

(Brief) Overview of the study >> Participant knows study

- Interview to capture the subjective experience of the physicians participating in the pilot project and to compare it to conventional consults
- Recording of the interview
- Voluntary participation/withdrawal anytime possible
- Preparation of a transcript. Analysis of pseudonymized data, so that no conclusions can be drawn about the participant during analysis and upon publication
- Passages can be removed upon request at any time, even afterwards
- Ask participant to sign a written consent form
- Thank for participation

Themes:

1. Consultation requests

2. Interdisciplinary rounds

3. Comparison of interdisciplinary rounds and consultation requests

Opening / career path:

“When I ask you questions, you will have as much time as you need to answer them. I will listen to you first and write down notes with regards to the different aspects, which I might come back to later. The aim is to capture your personal experiences and thoughts, and there is no right or wrong answer. We don't know each other yet, maybe you can tell me a few things about yourself, what has been your career path so far?”

Consultation requests:

“Do you have an example from the everyday life on the floor?

“What would you expect from other medical specialties regarding consults?”

“You mentioned that (X). Can you imagine that this would have turned out differently through interdisciplinary rounds?”

“What impact do consultation services have on workflow on the ward? Please tell me.”

Follow-up questions (by requesting details or paraphrasing), e.g.:

“I would like to go back to the notes I took.”

“You mentioned that... (X). Could you explain it in more detail?”

“You mentioned that... (X). Could you give some more examples?”

“You mentioned the circumstance X. Could you explain it in more details again?”

Interdisciplinary rounds / pilot project

“I would like to talk about the time before and after the implementation of the pilot project. When you think about personal experiences with consults in general, regardless of the medical specialty, what comes to your mind spontaneously?”

“Do you have an example from your everyday work?”

“What are your experiences with consultation requests for multimorbid patients?” (Definition: more than one chronic illness)

“You mentioned that (X). Can you imagine that this would have turned out differently through interdisciplinary rounds?”

“What was your assessment of the interdisciplinary rounds during the months they took place?

“To what extent was the response on the floor positive or negative (regarding the project)?”

“In your opinion, what influence did the interdisciplinary visits have on patient care and workflow on the floor? Please tell me.”

“How do you assess the interdisciplinary rounds in relation to your everyday work?”

“Did they lead to saving or increasing workload?”

“A study on the multidisciplinary care of internists and vascular surgeons on a vascular surgery ward came to the result that joint treatment improves the quality of care. To what extent, if at all, do you feel that regular joint rounds provide a benefit in the care of your patients?”

“Have you had any experience with interdisciplinary rounds outside of the pilot project? Please describe your experiences.”

Follow-up questions: like above.

Comparison of interdisciplinary rounds and consultation requests:

“Now, when you compare traditional consults with regular interdisciplinary rounds, what are your thoughts?”

“How do you evaluate the effectiveness of interdisciplinary visits compared to consults in terms of patient care, for example in the case of multimorbid patients?”

“How do you evaluate consults and the pilot project in terms of personal workload?”

“Keyword: employee satisfaction. What comes to your mind in light of what we just discussed?”

“If you could permanently change something about interdisciplinary collaboration, what would it be?”

If not mentioned yet:

“Which model would you prefer in the future? Why?”

“Did anything feel missing during the interdisciplinary rounds, or would you do anything differently in the future?”

Conclusion:

“Has anything else come to your mind in conjunction with interdisciplinary rounds or consult requests that we haven't discussed yet?”

“I thank you for your time and effort.”
