# Supplementary material for: Asking the generalist – evaluation of a General Practice rounding and consult service
Source: BMC Prim Care. 2024 Apr 16;25:113. doi: 10.1186/s12875-024-02353-0 (PMC11020190; doi:10.1186/s12875-024-02353-0)
Supplement: Supplementary file 6 — Supplementary Material 6 [file 12875_2024_2353_MOESM6_ESM.docx]

**APPENDIX**

Figure S1

Interview guideline G1-G4

COREQ-Checklist

*Figure S1: Sub-categories of challenges of conventional consults*
